# Storage duration of human blood samples for fatty acid concentration analyses - How long is too long?

**DOI:** 10.1016/j.mex.2024.102564

**Published:** 2024-01-13

**Authors:** Ghaith Mohsen, Helga Peisker, Katharina Gutbrod, Christian Stoppe, Georg Daniel Duerr, Markus Velten

**Affiliations:** a Department of Anesthesiology and Intensive Care Medicine, University Hospital Bonn, Germany; b Institute of Molecular Physiology and Biotechnology of Plants, University of Bonn, Bonn, Germany; c Department of Anesthesiology, Intensive Care, Emergency and Pain Medicine, University Hospital Würzburg, Würzburg, Germany; d Department of Cardiac Anesthesiology and Intensive Care Medicine, Charité Berlin, Berlin, Germany; e Department of Cardiovascular Surgery, University Medical Center Mainz, Germany; f Department of Anesthesiology and Pain Management, University of Texas Southwestern Medical Center, Dallas, TX, USA

**Keywords:** Fatty axcids, Docosahexaen acid, Clinical research, *Storage duration human blood FA concentration*

## Abstract

Polyunsaturated fatty acids such as DHA have known anti-inflammatory properties. The therapeutic implication highlights the importance of accurate serum measurements. Sample preservation is challenging when performed parallel to the clinical obligations. Impact of time between sample collection and processing regarding concentration alterations of fatty acids in human blood remains to be elucidated. Therefore, more information is required with respect to the stability and storage options in the context of potential degradation and concentration changes. This study investigates the stability of DHA in serum samples over time, given the challenges of timely sample analysis in clinical settings. Blood samples from three patients were collected and stored at +4 °C. Concentrations were analysed between 6 h and 7 days post-collection. Our data indicate that DHA concentrations remained unchanged during the observational period. Our results suggest that storage duration up to 7 days before sample processing does not affect accuracy of the results. DHA measurements is crucial for ongoing and future research in cardiovascular and inflammatory diseases. Our results reveal that DHA stability remains consistent over one week. This information is important for further clinical studies investigating PUFA concentrations, providing researches the option to postpone processing of samples if required along the clinical obligations.

## Graphical abstract

**Figure:**
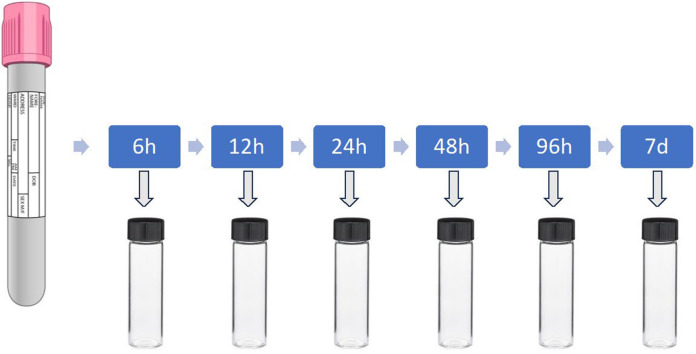

Specifications table

**Table:** 

Subject area:	Medicine and Dentistry
More specific subject area:	*Clinical research*
Name of your protocol:	*Storage duration human blood FA concentration*
Reagents/tools:	*N.A.*
Experimental design:	*DHA was isolated and analyzed from blood samples over a period of 7 days*
Trial registration:	*Rheinische Friedrich-Wilhelms-University Bonn, Germany, (protocol number 011/13)*
Ethics:	*Participants were included into the study after informed consent has been provided*
Value of the Protocol:	*This is the first study revealing DHA stability in human serum remains consistent over one week after withdrawal if serum samples are stored at +4* °*C.*

## Methods details

### Context and significance

Conducting molecular biological research using patient specimen is gaining more and more interest to allow personalized treatment approaches. Yet, there exist distinctive challenges, particularly when these samples are collected alongside routine clinical procedures. These obstacles encompass ensuring patient safety and complying with ethical and regulatory standards, akin to all clinical investigations. Nevertheless, a primary hurdle in the realm of molecular biological research alongside clinical responsibilities is the preservation of sample quality. Prolonged storage periods potentially impact the integrity of specimens if they cannot be processed promptly following collection.

Compliance with local, national, and international regulations is essential. Ethical considerations and informed consent processes are crucial in the perioperative setting. Patients or their legally authorized representatives should be fully informed about the research, including its potential risks and benefits. However, this is difficult if a study focusses on emergency procedures and enrolls patients that are unable to consent, but special regulations for these situations including waiver and consent after the procedure exist. This often leads to the problem, that samples must be stored and be protected from degradation until patients can give their consent or ethical concerns regarding the study are solved. If all is clarified, the samples can be released for further scientific investigations. Further, maintaining sterility is of utmost importance to prevent contamination of the collected specimens. Any breach in sterility could compromise research results. Thus, it is a major task to include specimen collection into the clinical process without interfering with institutional standards.

Polyunsaturated fatty acids (PUFAs) are fundamental in modulating inflammatory pathways via various mechanisms [1,2]. They are essential components of cellular membranes, and fluctuations in concentrations can profoundly influence both cellular signaling and membrane biophysical properties [3]. Specifically, docosahexaenoic acid (DHA) has been identified as key nutrient involved in modulating immune functions [4]. Its anti-inflammatory and anti-oxidative properties, including the inhibition of endotoxin-induced production of IL-6, TNF-α, and F2a-isoprostanes has been reported [3,5,6]. Furthermore, DHA exert its anti-inflammatory properties by lowering the synthesis of eicosanoids that originate from arachidonic acid (AA). This is achieved by competitively inhibiting the incorporation of AA into cellular membrane phospholipids, consequently reducing AA concentration within these membranes and by sequestration of pathway enzymes (desaturases and elongases involved in long-chain polyunsaturated fatty acid biosynthesis) that are shared by the AA and DHA synthesis pathways [7], [8], [9]. Additionally, studies indicate that DHA is linked to a suppressed activation of the proinflammatory transcription factor NF-κB (nuclear factor k-light-chain-enhancer of activated B cells) upon exposure to inflammatory stimuli. This suppression is attributed to the prevention of phosphorylation of I-κB, the inhibitory subunit of NF-κB [9,10].

Nevertheless, the exact roles and implications of DHA in clinical settings warrant further investigation [11]. As such, precise quantification of DHA concentration in human serum becomes imperative, not only to discern its physiological significance but also to comprehend the broader therapeutic effects they might possess in different clinical settings. The prompt analysis of DHA in serum immediately after sample withdrawal presents a significant logistical challenge, particularly given the limited timeframe and availability of methods to timely measure these in hospital setting. Instant serum analysis after sample collection is often not feasible, necessitating extended preservation and storage durations. This raises concerns regarding the stability of fatty acid concentrations in stored blood samples.

In view of these challenges, together with the cardinal role of DHA in health-related research, there emerges an urgent necessity to investigate the potential effects of delayed analysis on serum DHA concentration. Addressing this concern ensures that interpretations related to DHA concentrations, both in clinical and research domains, are precise, consistent, and indicative of genuine physiological states.

### Study design

• Sample Collection:

In accordance with the declaration of Helsinki, after the ethics approval by the institutional revenue board (IRB) at the Rheinische Friedrich-Wilhelms-University Bonn, Germany, (protocol number 011/13) and signed informed consent, six individual whole blood samples from three different patients scheduled for coronary artery bypass grafting were collected using aspiration technique in standard serum tubes (S-monovette 01.1601, Sarstedt AG & Co. KG, Germany) after induction of anesthesia and prior to surgery according to current guidelines and institutional standards for puncture of peripheral veins to avoid contamination of the samples. After collection the whole blood was allowed to clot in the s-monovette for 20–30 min at room temperature. Subsequently, s-monovettes were stored at +4 °C until further isolation and analysis.

• Time Points for Analysis:

To assess the stability of DHA over time, clotted samples were stored in serum monovettes at +4 °C until being processed after different durations (6 h, 12 h, 24 h, 48 h, 96 h, and 7 days). Subsequently, monovettes were centrifuged at 2000 g for 10 min, fatty acids were isolated, aliquoted into labeled cryovials, and stored at a −80 °C temperature to minimize degradation and to maintain DHA stability until fatty acid quantification.

• Lipid Isolation:

For lipid extraction, 200 µl of serum was transferred to 6 ml glass vials. After adding 1 mL methanol and 0.5 mL chloroform, 100 µl of pentadecanoic acid in methanol is introduced. The mixture underwent 20-minute-centrifugation to sediment cell debris, and the supernatant (termed "extract 1″) is stored. The pellet was then successively treated with two chloroform/methanol mixtures, centrifuged after each, and the resulting supernatants ("extract 2″ and "extract 3″) were combined with the initial extract and stored at −20 °C.

• DHA Quantification:

Upon reaching each time point, the respective samples were thawed at room temperature to ensure uniformity in handling. The isolated lipids were hydrolyzed and the fatty acids were converted to their methyl esters by treatment with 1 N methanolic HCl for analysis with gas chromatography (GC). Fatty acid methyl esters were detected using a flame ionization detector (FID), the quantification of DHA was based on comparison to the amount of the internal standard pentadecanoic acid [12].

• Data Analysis:

The DHA concentration for each time point was measured. We conducted a Friedman test comparing the values at each time point to the value at the first time point (6 h). The written code was instructed to do a Wilcoxon Test in case of a significant finding [12].

**Fig. 1:**
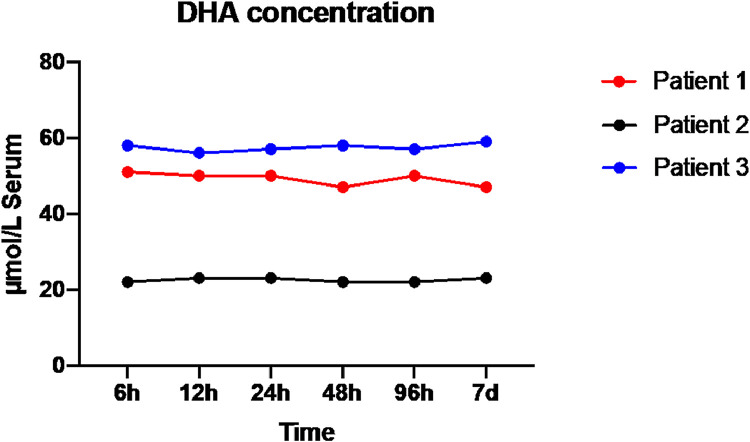
Time-course of DHA serum concentrations.

• Results:

As shown in Fig. 1, there were significant differences in DHA concentrations between individuals. However, individual DHA concentrations remained unchanged if serum samples were stored for a duration of up to seven days at +4 °C before fatty acids were isolated. Statistical analyses using Friedman test on the data set showed a p-value = 0.97.

### Study implication

The present study is investigating changes of DHA concentrations in human blood samples that were stored at +4 °C over a period of one week before samples were centrifuged and further processed for fatty acid isolation and concentration measurements.

Our data indicate that, while there were significant interindividual differences in DHA concentrations between patients, concentrations remained stable over a time period of one week when after blood draw samples continued in conventional tubes and were stored in a standard freezer without centrifugation or further processing. This information is of utmost importance to clinical scientist. Conduction clinical studies is becoming more challenging with increased clinical work load and limited research resources. Of note, generation preliminary data is at the forefront of every investigation potentially resulting in a successfully funded application and published project. However, financial research resources are limited at the begin and integrating the project into the clinical day to day work utilizing fewest resources is crucial. The herein reported information provides clinician scientist more flexibility integrating the collection and further processing of human samples into their clinical practice.

The examination of DHA stability in serum samples over various time intervals post-withdrawal is ensuring the integrity and accuracy of DHA measurements and not just essential for clinical evaluation but also pivotal as we base for future clinical research. The information that serum samples can be stored over a duration of 7 days at 4 °C without changes in concentrations of fatty acids has profound implications on clinical research and will allow implementing clinical research protocols and help clinician scientist to investigate fatty acid metabolism during their clinical obligations. Data received from this observational study might allow and encourage more research in this area to allow more personalized approaches in future.

## CRediT authorship contribution statement

**Ghaith Mohsen:** Investigation, Writing – original draft. **Helga Peisker:** Data curation, Formal analysis. **Katharina Gutbrod:** Formal analysis, Writing – review & editing. **Christian Stoppe:** Conceptualization. **Georg Daniel Duerr:** Conceptualization, Resources. **Markus Velten:** Writing – review & editing, Project administration.

## Declaration of competing interest

The authors declare that they have no known competing financial interests or personal relationships that could have appeared to influence the work reported in this paper.

## Data Availability

Data will be made available on request.
